# Implementing exercise programs to prevent falls: systematic descriptive review

**DOI:** 10.1186/s40621-016-0081-8

**Published:** 2016-07-04

**Authors:** Victoria Shier, Eric Trieu, David A. Ganz

**Affiliations:** 1 Pardee RAND Graduate School, RAND Corporation, 1776 Main St, Santa Monica, CA 90407 USA; 2 Cedars-Sinai Medical Center, 8700 Beverly Blvd, Los Angeles, CA 90048 USA; 3 VA Greater Los Angeles Healthcare System, David Geffen School of Medicine at UCLA, RAND Corporation, 11301 Wilshire Blvd., Building 158, Room 128, Los Angeles, CA 90073 USA

**Keywords:** Fall prevention, Exercise program, Descriptive systematic review

## Abstract

**Background:**

The United States Preventive Services Task Force recommends exercise to prevent falls in community-dwelling adults aged ≥ 65 years at increased fall risk. However, little is known about how best to implement exercise programs in routine care when a patient’s need for exercise is identified within the healthcare system.

**Methods:**

Using a qualitative approach, we reviewed the literature to determine how exercise programs to prevent falls are implemented from the vantage point of a health care setting. We synthesized descriptive information about each program with data on program features and implementation difficulties and facilitators.

**Results:**

We found that programs sponsored by primary care providers (PCPs) or specialists may help with recruitment into exercise programs. PCPs have the opportunity to identify people at risk and promote participation since most older adults regularly visit, and inquire about exercise from, their physicians. In terms of referral options, both home-based and group-based exercise programs have been shown effective in preventing falls; however, each approach carries strengths and limitations. Home-based programs can include participants who are reluctant or unable to attend group classes and can be individually tailored, but provide less opportunity for supervision and socialization than classes. Adherence to programs can be encouraged, and attrition minimized, through positive reinforcement. Successful programs ranged in expense for exercise sessions: a weekly class combined with exercises at home cost < $2 per participant per week, while frequent individual sessions cost > $100 per participant per week.

**Conclusions:**

With increasing attention to population-based health management in the United States, clinicians and health system leaders need a deeper understanding of how to link patients in their healthcare systems with appropriate community programs. This review identifies key characteristics of successful fall prevention exercise programs that can be used to determine which local options conform to clinical evidence. In addition, we highlight tradeoffs between program options, such as home versus group exercise programs, to allow referrals to be tailored to local conditions and patient preferences. Finally, our work highlights the key role of the PCP in recruiting patients to participate in exercise programs, and identifies options, such as registries, to support referrals to the community.

## Review

### Background

More than one-third of adults age ≥ 65 fall each year (Rubenstein and Josephson 2002; Tinetti 2003). Many people who fall suffer injuries such as lacerations, hip fractures, and head trauma (Stevens et al. 2006a) and may experience reduced mobility and physical fitness. Falls are the leading cause of injury-related deaths among adults age ≥ 65 (Centers for Disease Control and Prevention, 2016). The resulting hospitalizations, emergency department visits, and other treatments from falls are costly. Adjusted for inflation the annual direct medical cost of falls in the U.S. has been estimated at $34 billion (Centers for Disease Control and Prevention, 2016, Stevens et al. 2006b).

Several systematic reviews have demonstrated that exercise programs are effective in reducing falls and fall-related injuries in community-dwelling elders (El-Khoury et al. 2013; Gillespie et al. 2012; Michael et al. 2010; Sherrington et al. 2011; Sherrington et al. 2008). In addition, based on its own review of the evidence, the United States Preventive Services Task Force (USPSTF) recommended exercise or physical therapy as a primary care-relevant intervention to prevent falls in community-dwelling adults age ≥ 65 who are at increased risk for falls (Moyer 2012). However, despite the strong evidence base supporting the effectiveness of exercise programs to prevent falls, surprisingly little is known about how best to implement an exercise program when a patient’s need for exercise is identified in a health care setting, as noted in a commentary by Tinetti and Brach (2012). Such information is needed by primary care providers interested in implementing or referring patients to exercise-based fall prevention programs and to inform potential coverage determinations and implementation strategies by the Centers for Medicare and Medicaid Services (CMS) and commercial health plans. We hypothesized that although the current evidence base is organized around effectiveness, we could glean critical information to guide implementation of exercise programs using a qualitative approach to literature review (Centre for Reviews and Dissemination 2009). In this article we detail our findings from the review, and then focus on the implications for integration of exercise programs for prevention of falls with existing healthcare delivery systems.

### Methods

Seven relevant and recent systematic reviews of fall prevention interventions (Beswick et al. 2008; El-Khoury et al. 2013; Gates et al. 2008; Gillespie et al. 2012; Michael et al. 2010; Sherrington et al. 2011; Sherrington et al. 2008) served as source material for our analysis. All potential studies included in these systematic reviews, hereafter referred to as source articles, were assessed by two reviewers to determine whether they met the following criteria: (1) enrolled independently-living adults of mean age ≥ 65 years; (2) structured exercise or physical therapy was the only or core component of the intervention (all treatment participants needed to receive the exercise component for exercise to be a core component); (3) participants were recruited from, or the program was administered from a health care setting (defined as a location where a trained healthcare professional practices either on site or in the vicinity); (4) study design was a randomized controlled trial (RCT) and control group did not receive exercise; (5) study reported number of falls over a given period of time, number of people who fell over a given period, or time to first fall, and (6) study was reported in English. We limited our search to RCTs to focus on data derived from the same studies that have informed current guidelines. Differences regarding inclusion/exclusion of articles were resolved by consensus.

To augment the data provided in the original studies, we searched for related articles by reviewing source article bibliographies and searching for articles citing the source article in Web of Science and Google Scholar. The goal of searching for related articles was to find process evaluations that documented and evaluated implementation steps in detail.

Data were extracted by two reviewers using a standardized form. Discrepancies were resolved by discussion, with involvement of a third reviewer if necessary. The studies were described in terms of patient fall risk (above-average versus average/below-average), presence of exercise components using the taxonomy developed by Prevention of Falls Network Europe project (Lamb et al. 2011), aspects of the exercise program (setting, amount of supervision, progression of exercises, intensity of exercise), and intervention success. The study sample was classified as at above-average risk for falls if the baseline annualized probability of at least one fall was higher than 36 % in the sample, which is the upper limit of the 95 % confidence interval for the probability of falling at least once in any given year for an unselected sample of individuals age ≥ 65 (Ganz et al. 2007). If baseline data were not presented, we used the annualized probability of at least one fall among the control participants during the study period. We classified studies as successful if the pooled estimate of the effect of exercise on the rate of falls was below 0.91 (the upper limit of the 95 % confidence interval of the pooled estimate of the effect in a recent meta-analysis of exercise interventions; pooled rate ratio 0.84, 95 % CI 0.77–0.91; (Sherrington et al. 2011)). We estimated costs for exercise instruction per participant per week by multiplying the time of supervised exercise per week by the Bureau of Labor Statistics mean wage estimate in 2015 U.S. dollars for the instructor’s occupation (US Department of Labor, 2016). Quality of the RCTs (Table 2) was assessed by examining the Physiotherapy Evidence Database (PEDro) scale (www.pedro.org.au) which assesses the RCT on 10 criteria including random allocation, concealed allocation, and blinding of subjects, therapists, and assessors.

The research team identified the main categories important for implementation (e.g., recruitment, participant adherence, participant attitudes, implementation successes, difficulties and facilitators) and extracted the relevant qualitative data. This qualitative data was further coded using software (MAXQDA 10; Berlin, Germany) to identify themes within these categories.

## Results

### Study characteristics

Of the 128 relevant studies included in the seven systematic reviews, 29 met inclusion criteria. Seven were included in El-Khoury et al. 2013; 24 were included in Gillespie et al. 2012; 12 were included in Michael et al. 2010; 17 were included in Sherrington et al. 2008; 22 were included in Sherrington et al. 2011; and 1 came from our personal files. 22 of these were included in multiple systematic reviews. An additional 32 related articles that provided contextual information on the included RCTs were identified. Studies were excluded for the following reasons: 1 was not published in English, 41 studies did not include exercise or physical therapy as a core component, 3 were not RCTs, 1 study’s control group received exercise, 19 did not include independently-living adults with a mean age ≥ 65 years, 4 did not report falls outcome data, and 30 did not derive from a health care setting.

Studies took place in the following countries: Australia, Brazil, Canada, Chile, Finland, Germany, Japan, the Netherlands, Sweden, Switzerland, UK, and US. Table 1 provides a description of the participants, type of exercise, exercise setting, and instructors for successful and unsuccessful studies. Twenty studies were successful; nine were unsuccessful. Most included studies focused on a population at above-average risk for falls including patients with Parkinson’s disease or recent history of injurious falls. Physical therapists provided exercise instruction and supervision in a majority of programs (Ashburn et al. 2007a; Campbell et al. 1999b; Campbell et al. 1997; Campbell et al. 2005; Clemson et al. 2012; Green et al. 2002; Haines et al. 2009; Hornbrook et al. 1994; Kronhed et al. 2009; Latham et al. 2003a; Lin et al. 2007; Luukinen et al. 2007; Madureira et al. 2007; Protas 2005; Steadman et al. 2003; Swanenburg et al. 2007). Tai chi or exercise instructors provided instruction in three studies (Barnett et al. 2003; Dangour et al. 2011; Li et al. 2005; Logghe et al. 2009) and nurses supervised in two studies (Ebrahim et al. 1997; Robertson et al. 2001).

**Table 1:** Study characteristics

	All studies (*n* = 29)
Population	
Average age of participants (years)	74
% women	66 %
Above-average risk population, *n* (%)^a^	22 (76 %)
Type of exercise	
Gait, Balance, and functional training, *n* (%)	21 (72 %)
Strength/resistance, *n* (%)	21 (72 %)
General physical activity, *n* (%)	12 (41 %)
Flexibility, *n* (%)	8 (28 %)
Endurance, *n* (%)	6 (21 %)
3D (Tai Chi, Qi Gong, Dance), *n* (%)	3 (10 %)
Other, *n* (%)	1 (3 %)
Setting	
Home, *n* (%)	9 (31 %)
Class, *n* (%)	8 (28 %)
Class and home, *n* (%)	5 (17 %)
Clinic, hospital, other, *n* (%)	7 (24 %)
Instructor	
Physical therapists, *n* (%)	16 (55 %)
Tai chi or exercise instructor, *n* (%)	4 (14 %)
Nurse, *n* (%)	2 (7 %)
Other, *n* (%)	7 (24 %)

^a^Above-average risk population: Study sample classified as at above-average risk for falls if baseline annualized probability of at least one fall was higher than 36 % in the sample. This is the upper limit of the 95 % confidence interval for the probability of falling at least once in any given year for an unselected sample of individuals age ≥ 65 (Ganz et al. 2007)

We have organized key lessons about exercise programs into two major categories: program design and implementation (patient population; recruitment; type of exercise; tailoring to individuals; location; supervision; exercise frequency and program duration; and participant adherence during the program), and sustainability and scalability (program attrition; maintaining participant exercise after intervention; and resource implications).

### Program design and implementation

#### Patient population

For older adults identified through a healthcare setting, authors of several studies suggested that frail older people have the most to gain from interventions to reduce falls (Campbell et al. 1997; Carter et al. 2002; Robertson et al. 2001). For example, a meta-analysis of studies implementing the Otago Exercise Program, a home-based program of balance and strength training and walking, found the program is “significantly more effective in reducing the rate of fall injuries in those aged 80 and older than in younger trial participants… The program may be most effective in frailer, older people because the exercises increase strength and balance above the critical threshold needed for activities such as rising from a chair and going up and down the stairs and over home hazards (Robertson et al. 2002).” However, authors of studies also suggested that programs may experience less withdrawal among healthier patients (Logghe et al. 2011) and changing behaviors of very frail patients may be challenging (Ashburn et al. 2007a). While not focused on patients in the health care setting, a recent review found no difference in the effectiveness of exercise interventions delivered to the general community and to individuals with elevated risk (Gillespie et al. 2012).

#### Recruitment

The most common recruitment strategy included an initial letter from primary care providers (PCPs) (Table 2). Authors of three studies reported that having a program sponsored by the primary care practice or specialist may help with recruitment (Ashburn et al. 2007a; Barnett et al. 2003; Campbell et al. 1997). PCPs have the opportunity to identify people at risk and promote participation since most older adults visit their PCP at least once each year, seek advice about exercise from their physicians, and are more likely to participate in exercise if recommended by their physician (Barnett et al. 2003). However, as discussed in some studies, the success of these recruitment efforts may depend on the motivation and enthusiasm of the health professionals (Ashburn et al. 2007b; Campbell et al. 1997). Another potential recruitment method was the “use of similarly aged persons for personal contact with participants… [this] appealed to the credibility and sense of ownership for the participants (Stevens et al. 1992).”

**Table 2:** Summary of exercise programs for included studies

Authors (year) Successful/Unsuccessful^a^ Quality Score (of 10)^b^	Exercise setting; Standardized (S), Individualized (I), Semi- individualized (SI)	Population and recruitment	Above-average risk or Average/below-average risk^c^	Exercise description and Instructor	Supervised/Unsupervised: Exercise components^d^ Time, Frequency and Program Duration	Adherence	Attrition rate	Exercise instruction cost per participant^e^
Ashburn et al. (2007a) Successful (Albanese 2007; Ashburn 2007b; Ashburn 2008) 8	Home; I	Patients with idiopathic Parkinson’s disease with more than 1 fall in past year identified through clinical registers of specialists; letter sent	Above-average Risk	Treatment goals established and exercises from exercise menu were taught in home. Menu designed with 6 levels of progression and comprised muscle strengthening, range of movement, balance training and walking. Exercises chosen at appropriate level and progressed at each visit. Patients encouraged to continue exercise after 6 weeks. Instructor: physiotherapist	Supervised: Balance, Strength, Flexibility, General physical activity • 1 hr, 1 time per week, 6 weeks Unsupervised: Balance, Strength, Flexibility, General physical activity • Daily	69 of 70 had at least 6 sessions	2 % per month	$41per week
Barnett et al. (2003) Successful 8	Class and Home; S	Women and men aged 65 years and older recruited from general practice clinics or acute hospital physiotherapy departments	Above-average Risk	Group exercise class to improve balance, coordination, aerobic capacity and muscle strength. Participants also received a home exercise program based on class content. Also received information on practical strategies for avoiding falls. Instructor: accredited exercise instructor	Supervised: Strength, Balance, Endurance, Flexibility, 3D • 1 hr, 1 time per week, 1 year Unsupervised: based on class content but not specified	Participants attended median of 23 classes; 91 % of those who attended classes performed home exercises 1+ times per week	2 % per month	$2 per week
Buchner et al. (1997) Successful (Buchner et al. 1993a; Buchner 1993b; Ory et al. 1993) 7	Class; S	Women and men aged 68 to 85 years old with mild deficits in strength and balance enrolled in HMO; PCP approved participation and sent letter	Average/below-average Risk	Exercise consisted of endurance training and/or strength training in supervised classes. Exercise sessions began with a 10- to 15-min warm-up and ended with a 5- to 10-min cool-down. Endurance training used stationary cycles. Strength training groups did resistance exercise of the upper and lower body using weight machines. Subjects received a discharge planning intervention to promote continued exercise. Instructor: NR	Supervised: Strength, Endurance • 1 hr, 3 times per week, 24-26 weeks	Participants attended 95 % of scheduled exercise sessions	3 % per month	$8 per week
Bunout et al. (2005) Unsuccessful 5	Class; SI	Elderly women and men; recruitment not reported	Average/below-average Risk	Moderate-intensity resistance exercise training program included functional weight bearing exercises, squats, step-ups in stair, and arm pull-ups. Participants also engaged in walking before and after resistance training. Instructor: specialized coach	Supervised: Strength, General physical activity • 1 hr, 2 times per week, 1 year	Participants attended 52 % of sessions	NR	$3 per week
Campbell et al. (1997, 1999a) Successful (Campbell and Robertson 2010; Campbell and Robertson 2007; Gardner et al. 2001; Robertson and Campbell 2008; Robertson et al. 2002) 8	Home; I	Women aged 80 and older identified from registers of general practices; invited by PCP to participate	Above-average Risk	Otago: Physiotherapist prescribed exercises individually during 4 visits over first 2 months. Program consisted of muscle strength, balance retraining, and walking program. Level of difficulty increased by increasing number of repetitions and weights. Instructor: physiotherapist	Supervised: Balance, Strength • 1 hr, 4 times over 1st 2 months Unsupervised: Balance, Strength • 30 min, 3 times per week, 1 year Unsupervised: General physical activity • 2 times per week, 1 year	At end, 44 % of total were still exercising 3+ times per week	4 % per month during second year	$20 per week
Campbell et al. (1999b) Successful (Campbell and Robertson 2010;Campbell and Robertson 2007; Gardner et al. 2001; Robertson and Campbell 2008; Robertson et al. 2002) 6	Home; I	Women and men aged 65 and older taking antidepressant or tranquilizer identified from register of general practice groups; invited by PCP	Above-average Risk	Otago: Physiotherapist prescribed exercises individually during 4 visits over first 2 months. Program consisted of muscle strength, balance retraining, and walking program. Level of difficulty increased by increasing number of repetitions and weights. Also included Medication withdrawal component. Instructor: physiotherapist	Supervised: Balance, Strength • 1 hr, 4 times over 1st 2 months Unsupervised: Balance, Strength • 30 min, 3 times per week, 44 weeks Unsupervised: General physical activity • 2 times per week, 44 weeks	After 44 weeks, 63 % of those remaining completed their exercise 3+ times per week, 72 % walked 2+ times per week	5 % per month	$20 per week
Campbell (2005) Unsuccessful (Campbell and Robertson 2010; Campbell and Robertson 2007; Gardner et al. 2001; Robertson and Campbell 2008) 8	Home; I	Women and men aged 75 years and older with poor vision from register, optometry clinic, and low vision outpatient clinics	Above-average Risk	Otago: Physiotherapist prescribed exercises individually during 5 visits over 6 months. Program consisted of muscle strength, balance retraining, and walking program. Level of difficulty increased by increasing number of repetitions and weights. Program was modified for those with severe visual acuity loss. Instructor: physiotherapist	Supervised: Balance, Strength • 1 hr, 5 times over 6 months Unsupervised: Balance, Strength • 30 min, 3 times per week, 1 year Unsupervised: General physical activity • 2 times per week, 1 year	18 % completed exercises 3+ times per week, 36 % completed exercises 2 times per week. 44 % walked 2+ times per week	1 % per month	$8 per week
Carter (2002) Successful (Campbell 2002; Carter et al. 2001) 5	Class; S	Women with osteoporosis recruited from those diagnosed at health center	Above-average Risk	Osteofit targets posture, balance, gait, coordination, and hip and trunk stabilization with 8 to 16 strengthening and stretching exercises and strength training. Instructor: certified by hospital osteoporosis program	Supervised: Balance, Strength, Flexibility • 40 min, 2 times per week, 20 weeks	NR	4 % per month	$5 per week
Clemson et al. (2012) Successful (Clemson et al. 2010) 7	Home; I	Women and men aged 70 years or older with two or more falls or one injurious fall in past 12 months; recruited from Veteran’s Affairs and general practice databases	Above-average Risk	Lifestyle integrated Functional Exercise (LiFE) program included movements prescribed to improve balance or increase strength that are embedded within everyday activities so that movements can be done multiple times per day. Instructor: physiotherapist	Supervised: Balance, Strength • 7 times over 6 months Unsupervised: Balance, Strength • Multiple times per day, 7 days per week, 1 year	64 % completed exercises at 12 months; 3.89 mean days per week exercised in final month	2 % per month	$6 per week
Dangour et al. (2011) Unsuccessful (Dangour et al. 2007; Walker et al. 2009) 7	Class; SI	Women and men aged 65 to 67.9 recruited from health center catchment areas	Average/below-average Risk	Physical activity group training sessions focused on resistance exercises. Participants were encouraged to walk to sessions. Instructor: physical activity instructor	Supervised: Strength, General physical activity • 1 hr, 2 times per week, 2 years	38 % attended at least 24 classes over 12 months	1 % per month	$3 per week
Ebrahim et al. (1997) Unsuccessful 6	Home; SI	Women who had sustained an upper arm fracture in past 2 years from registers of Emergency Department and orthopedic clinics of hospitals; recruited through letters explaining study	Above-average Risk	Participants encouraged to gradually work up to walking for 40 min, 3 times a week. Instructed to progressively increase the amount and speed of walking. Seen every 3 months to discuss problems, reinforce intervention and allow physiological measurements to be taken. Instructor: nurse	Unsupervised: Endurance • 40 min, 3 times per week, 2 years	All who remained in trial reported regular walking	3 % per month during year 1	$3 per week
Freiberger et al. (2012) Successful (Fitness group) (Freiberger et al. 2007) 8	Class and Home; SI	Women and men aged 70+ who had fallen in past 6 months or fear of falling from health insurance company membership database; recruited through questionnaires	Above-average Risk	All 3 interventions included strength and balance exercises but differed regarding their second feature (Additional strength and balance; endurance; fall risk education). Interventions were progressive over time and included intro discussion, warm-up exercise, main program, cool-down, and discussion. For home, participants received brochure describing how to perform the strength, balance, and gait exercises. Instructor: Fall prevention instructors	**Strength and balance group:** Supervised: Balance, Strength • 1 hr, 2 times per week, 4 months Unsupervised: Balance, Strength • Daily **Fitness group:** Supervised: Balance, Strength, Endurance • 1 hr, 2 times per week, 4 months Unsupervised: Balance, Strength • Daily **Multifaceted group:** Supervised: Balance, Strength • 1 hr, 2 times per week, 4 months Unsupervised: Balance, Strength • Daily	82 % of strength and balance group, 84 % of fitness group, and 84 % of multifaceted group attended at least 24 of the 32 sessions	1 % per month	$6 per week
Green et al. (2002) Unsuccessful (De Weerdt and Feys 2002; Dowswell et al. 2002; Green et al. 2004) 8	Outpatient rehab center or Home; I	Women and men who had a stroke at least 1 year previously and an associated persisting mobility problem; recruited from hospital and community therapy stroke registers	Above-average Risk	Patients were assessed by a physiotherapist and then treated with a problem solving approach at home or in outpatient rehabilitation centers. The main interventions given were gait re-education, exercise therapy, functional exercises, and balance re-education Instructor: physiotherapist	Supervised: Balance • At least 3 contacts over 13 week period	Median: 3 treatments per patient	2 % per month	$7 per week
Haines et al. (2009) Successful 7	Home; SI	Women and men treated on geriatric rehabilitation, medical, or surgical units of hospital with gait instability; physiotherapist identified patients and referred to research team	Above-average Risk	First home visit provided instruction for the Kitchen Table Exercise Program. Program consisted of DVD and workbook for progressive exercise program combining lower limb strength and balance exercises. Program included 6 types of exercises each with 6 different levels of difficulties. Instructor: physiotherapist.	Supervised: Balance, Strength • At least initial and follow up visit Unsupervised: Balance, Strength • 2 times per week, 8 weeks	15 of 19 attempted the program at least once during week 1; 8 attempted 1+ times during week 8	0 % per month	$10 per week
Hauer et al. (2001) Successful 6	Class at outpatient rehab unit; SI	Women with fall as reason for admission to hospital or recent history of injurious fall; recruited at end of rehabilitation from hospital	Above-average Risk	Ambulatory training of strength, functional performance, and balance. Exercise included warm up on stationary cycles, high-intensity progressive resistance training of functionally relevant muscle groups, and training in walking, stepping, and sitting to modify unsafe or inefficient performance. Patients progressed to advanced levels of exercise. Physiotherapy included massaging, stretching, and heat/ice to orthopedic problems. Instructor: therapeutic recreation specialist.	Supervised: Balance, Strength • 2.25 hr, 3 times per week, 3 months Supervised: Flexibility, Other • 25 min sessions, 2 times per week, 3 months	85 % adherence	8 % per month	$50 per week
Hornbrook et al. (1994) Successful (Stevens et al. 1991; Stevens et al. 1992) 5	Class and Home; S	Members of large HMO aged 65 years and older; Letter sent with follow up phone call or home visit if no response	Average/below-average Risk	Weekly group meetings included didactic presentations, demonstrations of falls prevention exercises, and small group meetings. Exercises were chosen to provide active involvement of all body parts, maintain full range of motion of all joints, provide strengthening, improve posture by preventing forward flexion of the head and shoulders, and improve balance. Participants were given a manual describing the exercises to follow at home and encouraged to begin walking. After the first 4 sessions, quarterly maintenance sessions were held. Instructor: health behaviorist and physical therapist	Supervised: Balance, Strength • 90 min, 1 time per week, 4 weeks Unsupervised: Balance, Strength • Time not specified Unsupervised: General physical activity • 3 times per week	78 % attended at least 1 session, 61 % attended 3+ sessions	NR	$4 per week
Iwamoto et al. (2009) Successful 6	Clinic or hospital; S	Women and men aged 50 or older who visited hospital Department of Orthopedic Surgery or clinic	Above-average Risk	Exercise program in clinic or hospital consisted of calisthenics, body balance training, muscle power training, and walking ability training. Instructor: NR	Supervised: Balance, Strength, Flexibility • 30 min, 3 times per week, 5 months	Compliance with exercise 100 %	0 %	$62 per week
Kronhed et al. (2009) Successful (Kronhed 2009) 7	Class; I	Women with established osteoporosis and at least one fragility fracture identified from files at the Osteoporosis Unit at hospital; invitation letter sent	Average/below-average Risk	Exercise consisted of a strength training program supervised by a physiotherapist. The program consisted of a warm-up using exercise bicycles and a cross-trainer. Back strengthening exercises, abdominal muscle training, sequence training exercises, and balance exercises were performed. In the introductory instruction, participants received personal instruction and an individually designed load that was progressively increased according to the participant’s capacity. Sessions finished with 10 min of stretching. Participants were encouraged to continue the training exercise program on their own at senior gyms after the supervised group exercise training period. Instructor: physiotherapist	Supervised: Balance, Strength, Flexibility, Endurance • 1 hr, 2 times per week, 4 months	Participants completed an average of 24 of 30 sessions	5 % per month	$3 per week
Latham et al. (2003a) Unsuccessful (Judge 2003; Latham 2003b) 8	Hospital and Home; SI	Frail women and men aged 65 and older admitted to geriatric rehabilitation units	Above-average Risk	Resistance exercise consisted of a quadriceps exercise program using adjustable ankle cuff weights. The aim was for patients to exercise at a high intensity by midway through the program. Each session began with individualized warm-up stretches, followed knee extensions. Most of the patients performed their first two exercise sessions in the hospital and continued the rest of their sessions at home. Instructor: physical therapist	Supervised: Strength • 2 sessions, then biweekly Unsupervised: Strength • 3 times per week, 10 weeks	Patients adhered to 82 % of prescribed sessions	3 % per month	$29 per week
Li et al. (2005) Successful (Harmer and Li 2008; Li et al. 2004; Li et al. 2008) 5	Class; S	Women and men aged 70 years or older enrolled in non-profit hospital system; recruited through letter sent by PCP, follow-up call from research staff.	Above-average Risk	Classical Yang Style Tai Chi (24 forms) classes emphasize multidirectional weight shifting, awareness of body alignment, and movement coordination. Instructor: experienced Tai Chi instructor	Supervised: 3D • 1 hr, 3 times per week, 26 weeks	Median compliance was 61 of 78 sessions	5 % per month	$4 per week
Lin et al. (2007) Successful 5	Home; I	Women and men aged 65 and older who had medical attention due to a fall; recruited from clinics and hospitals	Average/below-average Risk	Exercise consisted of stretching, muscle strengthening, and balance training at increasing levels of difficulty. The training was individualized for each participant and consisted of 10 min of warm-up, 30 min of exercise, and 10 min of cool-down. Participants were instructed to practice these exercises at least three times a week. Instructor: physical therapist	Supervised: Balance, Strength, Flexibility • Every 2 weeks, 4 months Unsupervised: Balance, Strength, Flexibility • 40-60 min, 3 times per week, 4 months	NR	6 % per month	$17 per week
Logghe et al. (2009) Unsuccessful (Logghe et al. 2011; Zeeuwe et al. 2006) 8	Class and Home; S	Women and men aged 70 years and older; recruited from patient registration files of PCPs; letters sent by PCP	Above-average Risk	Tai Chi Chuan training with ten positions. Participants asked to practice the Tai Chi Chuan positions at home. Instructor: Tai Chi Chuan instructor	Supervised: 3D • 1 hr, 2 times per week, 13 weeks Unsupervised: 3D • 15 min, 2 times per week, 13 weeks	47 % attended at least 80 % of lessons, 85 % completed home exercise	9 % per month	$4 per week
Luukinen et a. (2007) Unsuccessful (Lehtola et al. 2006; Luukinen et al. 2006) 6	Class and/or home; I	Women and men aged 85 years or older; Recruitment not reported	Above-average Risk	Individual intervention plans were made during home visits by physiotherapist and occupational therapist based on risk factors. Home and group exercise, and walking exercises were recommended. Instructor: physiotherapist and occupational therapist	Supervised: General physical activity • Time not reported Unsupervised: not reported • 3 times per day, 1.5 years	NR	<1 % per month	NR
Madureira et al. (2007) Successful 6	Class and Home; S	Women aged 65 years and older; recruited from patients of Osteometabolic Disease Outpatient Clinic	Above-average Risk	Balance Training Program consisted of warm-up and stretching, walking, and balance training in dynamic and static positions. Patients were encouraged to continue same exercises at home. Instructor: experienced physiotherapist	Supervised: Balance, Flexibility, General physical activity • 1 hr, 1 time per week, 1 year (40 classes total) Unsupervised: Balance, Flexibility, General physical activity • 30 min, 3 times per week, 1 year	60 % participated in all classes; 77 % completed home exercises 1+ times per week	1 % per month	$1 per week
Protas et al. (2005) Successful 5	Outpatient research center; I	Men with idiopathic Parkinson’s disease diagnosed at Veterans Affairs center	Above-average Risk	Gait training consisted of walking on a treadmill at speed greater than over ground walking speed while walking in 4 directions and supported in harness for safety. Step training included suddenly turning treadmill on and off while subject stood in safety harness. Instructor: physical therapist	Supervised: Balance • 1 hr session, 3 times per week, 8 weeks	NR	NR	$124 per week
Robertson et al. (2001) Successful (Campbell and Robertson 2010; Campbell and Robertson 2007; Gardner et al. 2001; Robertson and Campbell 2008; Robertson et al. 2002) 8	Home; I	Women and men aged 75 and older from registers at 17 practices; letter sent from PCP	Average/below-average Risk	Otago: Set of muscle strengthening and balance retraining exercises that progressed in difficulty and a walking plan. Program individually prescribed during 5 home visits at weeks 1, 3, 4, and 8, and a booster visit after 6 months. Instructor: nurse	Supervised: Balance, Strength • 5 times Unsupervised: Balance, Strength • 30 min, 3 times per week, 1 year Unsupervised: General physical activity • 1 year	43 % exercised 3+ times per week, 72 % exercised 2+ times per week, 71 % walked 2+ times per week	1 % per month	$3 per week
Rubenstein et al. (2000) Successful 7	Class; S	Men aged 70 years or older with at least 1 key fall risk factor; recruited through Veterans Affairs Ambulatory Care Center.	Above-average Risk	Group exercise focused on increasing strength and endurance and improving mobility and balance. Strengthening exercises progressed over first 4 weeks. Endurance training included bicycle, treadmill, and indoor walking. Balance training increased in difficulty over 12 weeks. Instructor: exercise physiology graduate students.	Supervised: Balance, Strength, Endurance • 90 min, 3 times per week, 12 weeks	Participants attended 84 % of sessions	3 % per month	$10 per week
Steadman et al. (2003) Unsuccessful 5	Hospital; I	Subjects aged 60 and older recruited from attendees at multidisciplinary falls clinic	Above-average Risk	Assisted walking within parallel bars, assessment for mobility aids, stair practice, general bed mobility skills, and transfers. Balance exercises consisted of repetition of a series of graded tasks specific to functional balance with targets of distance and time to provide feedback. Also discussed fall avoidance behaviors and strategies for coping with a long time on the floor. Instructor: clinical physiotherapists	Supervised: Balance • 45 min, 2 times per week, 6 weeks	Random selection of patients showed protocol being adhered to	10 % per month	$62 per week
Swanenburg et al. (2007) Successful 6	Hospital physiotherapy dept.; I	Women with osteoporosis; recruitment not reported.	Above-average Risk	Program tailored to individual during initial 2 weeks. Two sessions per week focused on progressive resistance training and individual exercises to improve coordination, balance and endurance. One session per week consisted of a group exercise focused on balance exercises and games. Participants were also given daily protein supplements and encouraged to continue program after initial 3 months. Instructor: physical therapist	Supervised: Balance, Strength, Endurance • 70 min, 2 times per week, 12 weeks Supervised: Balance • 70 min, 1 time per week, 12 weeks	Compliance with exercise 93 %	3 % per month	$144 per week

*PCP* primary care provider, *NR* not reported, *HMO* Health Maintenance Organization

^a^ Successful/Unsuccessful: Study classified as successful if intervention results were within the pooled estimate of the effect of exercise on the rate of falls in the meta-analysis of exercise interventions (pooled rate ratio 0.84, 95 % CI 0.77-0.91) by Sherrington et al. 2011

^b^ Quality score: Quality of RCTs was assessed by Physiotherapy Evidence Database (PEDro)

^c^ Above-average risk/Average/below-average risk population: Study sample classified as at above-average risk for falls if baseline annualized probability of at least one fall was higher than 36 % in the sample. This is the upper limit of the 95 % confidence interval for the probability of falling at least once in any given year for an unselected sample of individuals age ≥ 65 (Ganz et al. 2007)

^d^ Exercise components: Balance: Gait, balance, and functional training; Strength: Strength/resistance; 3D: Tai Chi, Qi Gong, Dance

^e^ Exercise cost per participant calculated using the Bureau of Labor Statistics Occupational Employment and Wages, May 2015 for: Fitness trainers http://www.bls.gov/oes/current/oes399031.htm; Instructor not reported, median wage of other studies; Physical therapist http://www.bls.gov/oes/current/oes291123.htm; Recreation therapist http://www.bls.gov/oes/current/oes291125.htm; Registered nurse http://www.bls.gov/oes/current/oes291141.htm

Within people age ≥ 65, there were unique challenges to recruiting both younger and older subgroups. The youngest older adults were more likely to be busy with work or travel and the oldest were more likely to be ineligible because of physical limitations, medical problems, or institutionalization. Authors of studies reported that involving family members and PCPs in recruitment (Campbell et al. 1997) and offering free transportation might be particularly important for frail older adults (Stevens et al. 1992).

#### Type of exercise

The type of exercise in successful programs varied substantially (Table 2). Gait, balance, and functional training and strength/resistance training were the most common. Programs also targeted activities such as flexibility, general physical activity, endurance, and 3D exercise (Tai Chi, dance). Only five studies involved single components of exercise (Ebrahim et al. 1997; Li et al. 2005; Logghe et al. 2009; Luukinen et al. 2007; Steadman et al. 2003) and only one of these was successful (Li et al. 2005).

#### Tailoring program to individuals

Programs ranged from completely standardized to completely individualized, with intermediate strategies such as having standardized weight-bearing exercises with the progression to heavier weights tailored to each individual. One study tailored the program for each individual by suggesting strategies to improve balance that can be integrated into the participant’s everyday activities (Clemson et al. 2012). Authors of another study noted that, particularly among frail participants, “The program needs to be individually tailored because older people vary considerably in their physical capacity and health in response to exercise (Gardner et al. 2001).”

#### Location

Studies that included participants at above-average risk implemented exercise programs in a range of settings. Home-based programs typically involved the instructor making an initial visit to the participant’s home, followed by additional visits to increase the difficulty of the exercise or reinforce adherence. Exercise programs in the participant’s home had the advantage that they could be followed indefinitely (Campbell et al. 2002) and may be easier to maintain than group programs (Campbell et al. 1997). However, home-based programs offer less supervision (Haines et al. 2009). Authors of one study noted that the “use of high-intensity exercise in a home-based program might have had a greater risk of injuries and adverse events in these participants than a program of lower intensity…Most high-intensity programs for older people have been conducted under highly supervised conditions (Latham et al. 2003a).”

Exercise programs delivered in class settings have several advantages including social interaction (Carter et al. 2002; Hornbrook et al. 1994; Logghe et al. 2009), peer reinforcement and encouragement (Carter et al. 2002; Hauer et al. 2001; Hornbrook et al. 1994), and efficient use of instructor time (Carter et al. 2002; Hornbrook et al. 1994). However, some participants do not want to attend group exercise sessions, and illness may leave a participant unable to resume the exercises at the class level (Campbell and Robertson 2010).

Studies that combined class and home components included a class program with homework assignments or instructions for continuing exercises at home after the class ended.

#### Supervision

Most home-based programs used a combination of home visits and telephone calls to supervise participants. Instructors made weekly (Haines et al. 2009), monthly (Ashburn et al. 2007a; Ebrahim et al. 1997), or other regular (Campbell et al. 1999b; Campbell 1997) telephone calls once the home visits ceased, or made telephone calls during months with no scheduled home visit (Campbell et al. 2005; Robertson et al. 2001).

#### Exercise frequency and program duration

Among successful studies, total weekly exercise time ranged from 80 min (Carter et al. 2002) to more than 7 h (Table 2) (Hauer et al. 2001). Most successful studies prescribed exercise three or more times weekly. Authors of one unsuccessful study suggested that “lack of significant improvement in mobility of study participants could have resulted from too few treatments (Green et al. 2002).” As described by one successful program that prescribed 30 min sessions three times a week, programs should consider the population’s ability to sustain the intensity of exercise: “the intensity and frequency of the exercise program were considered to be reasonable for the elderly to be continued without any fatigue and difficulty for 5 months (Iwamoto et al. 2009).”

Duration of successful programs ranged from eight weeks to two years. Authors of a successful Tai Chi program noted: “our results indicate that several months of practice were needed before significant decreases in falls occurred, suggesting that no tangible results in falls should be expected from short-term exposure to Tai Chi (i.e., less than 3 months) (Li et al. 2005).” The duration may be particularly important for group exercise programs because “the benefit from the exercises lasts only for as long as the person participates in the program (Campbell 2002).”

#### Participant adherence

The level of adherence was highly variable (Table 2). For example, one study reported 18 % of participants completed all prescribed exercises in their home during the second year (Campbell et al. 2005) while another study reported 100 % adherence with supervised exercise sessions at a clinic or hospital for 5 months (Iwamoto et al. 2009).

Barriers to high adherence included unwillingness to change lifestyle and habits (Luukinen et al. 2007) and discomfort (Haines et al. 2009). For group exercises, having a pleasant and convenient location (Logghe et al. 2009; Madureira et al. 2007) and providing transportation or reimbursing travel costs (Hauer et al. 2001; Hornbrook et al. 1994; Logghe et al. 2009) can improve adherence. Authors of a successful class exercise program noted that “A secure environment, session supervision and the opportunity for social interaction reduce the feeling of isolation. A social support system is considered important in group activities, and helps sustain adherence and the effectiveness of the weekly exercise sessions and also the adherence to home-based exercises (Madureira et al. 2007).” The role of transportation, particularly for frail individuals, was described by authors of another successful study: “Relying on participants to provide their own transportation creates a strong selection effect towards persons with higher levels of functioning… Many of the participants aged older than 80 had stopped driving and were not comfortable using public transportation (Stevens et al. 1992).”

Studies also suggested that encouragement and supervision may improve adherence (Bunout et al. 2005; Campbell et al. 1997; Madureira et al. 2007). In classes, instructors can provide direct supervision of participants performing the exercises. Calls to individuals who miss classes can provide “active reinforcement to encourage participation in the program (Bunout et al. 2005).” For home-based exercises, providing participants with a manual of instructions and illustrations can improve adherence to the exercises prescribed (Madureira et al. 2007).

### Sustainability and Scalability

#### Program attrition

The median rate of attrition was 3 % per month (range: 0–10 %) (Table 2). The study with the highest attrition rate reported losing 65 of the 198 participants for the following reasons: new or worsening health problems (*n* = 29), moving away (*n* = 6), participant choice (*n* = 12), and unknown (*n* = 12) (Steadman et al. 2003). Authors of one study reported its relatively low attrition rate was due to “telephone calls to those who failed to attend more than two training sessions, which provided active reinforcement to encourage participation in the program (Bunout et al. 2005).”

#### Maintaining exercise after intervention

Several studies stated that participants remained active after the intervention (Barnett et al. 2003; Li et al. 2005; Swanenburg et al. 2007). One Tai Chi program indicated that more than half the participants remained physically active after the intervention and authors described the potential for convincing participants to make an active lifestyle change: “Tai Chi appears to be an appealing exercise program, with benefits that go beyond functional balance improvements and falls reduction, and that can be regarded as a self-regulated, enjoyable, and socially engaging activity (Li et al. 2004).”

#### Resource implications

The cost for exercise instruction varied based on whether the instruction was completed in a class setting versus one-on-one, the frequency and duration of supervised exercise, and the instructor qualifications. The estimated cost for instruction by a physical therapist of a successful program that provided weekly, one hour classes with 30 participants per class, supplemented with home exercises three times per week, was estimated at less than $2 per participant per week (Madureira et al. 2007). A second successful study had more frequent supervised exercise with three one-hour individual sessions provided per week at an outpatient research center (Protas et al. 2005). The estimated cost for this instruction is more than $100 per participant per week.

### Discussion

The USPSTF recommends exercise or physical therapy to prevent falls in community-dwelling adults age ≥ 65 years who are at increased risk for falls. Systematic reviews demonstrate that exercise programs are effective in reducing falls in community-dwelling elders (El-Khoury et al. 2013; Gillespie et al. 2012; Michael et al. 2010; Sherrington et al. 2011; Sherrington et al. 2008). Recent meta-analyses also examined whether different features of exercise programs are associated with greater falls prevention effects (Sherrington et al. 2011; Sherrington et al. 2008). While successful community-based fall prevention programs exist, additional efforts are needed to help integrate effective exercise programs with health care settings. This article builds on the previous work, using a complementary qualitative approach, and provides insight on how to implement exercise programs where participants are recruited from, or receive exercise in, a health care setting. As such, our findings are relevant to clinicians and health plan/integrated delivery system leaders who seek to strengthen their connections with exercise programs. Data from this review may be used to determine appropriate options for the types of exercise offered, location, frequency, duration, and type of supervision. In addition, we have synthesized information on approaches to maintaining adherence and reducing attrition, and have estimated some of the resource implications of program delivery.

Tinetti and Brach question whether all adults age ≥ 65 or only those with an elevated risk for injury should be encouraged to participate (Tinetti et al. 2012). Although most fall prevention programs are designed for older adults with an elevated risk for falls and fall injury, Sherrington et al. (2011) recommended that programs should target both the general community and those at high risk for falls, and we found five studies that implemented successful interventions for older adults without an elevated risk (Buchner et al. 1997; Hornbrook et al. 1994; Kronhed et al. 2009; Lin et al. 2007; Robertson et al. 2001). One of these studies (Hornbrook et al. 1994) decreased the odds of falling by 0.85 among independently living HMO members age ≥ 65. This program required limited resources for instruction and supervision by providing four initial weekly group meetings with up to 25 participants combined with exercises to follow at home. Quarterly maintenance sessions helped maintain participant motivation. To identify the targeted patient population, feasible screening strategies exist (Kenny et al. 2011; Reuben et al. 2003), and can be implemented particularly effectively in settings with an electronic health record (Spears et al. 2013; Wenger et al. 2010) or care management system (Wenger et al. 2011). Potential participants may then be offered exercise programs after PCP review for appropriateness, much as participants in some of the studies we reviewed were recruited using PCP registries.

In regards to the type of exercise that should be included in a program, Sherrington et al. (2011) found that that exercise including a moderate or high challenge to balance was more effective in reducing falls than exercise lacking this component. Our study was not designed to confirm these findings directly, but noted that exercise programs in health care settings typically had multiple components (gait/balance/functional training and strength/resistance training being the most common); of five studies that were limited to a single exercise type, only one was successful.

To encourage patient participation, exercise programs should highlight a range of benefits in addition to fall prevention (Tinetti et al. 2012). Many studies have found benefits across a greater range of outcomes including quality of life (Ashburn et al. 2007a; Lin et al. 2007), participation in activities of daily living (Lin et al. 2007), physical functioning (Barnett et al. 2003; Bunout et al. 2005; Li et al. 2005; Madureira et al. 2007), and pain (Kronhed et al. 2009). Physicians and health plans should tout these benefits during referral to exercise programs because older adults are motivated to participate by a wide range of perceived benefits such as enjoyment, improved health, mood, and independence (Yardley et al. 2006), and some older people do not perceive a need for a fall prevention program when advertised as such (Calhoun et al. 2011). Of note, Sherrington et al. (2011) recommended that programs may be delivered in a group or home-based settings and we found that both home-based and class-based exercise can be successfully implemented; the decision about which programs to offer may depend on geography (e.g., urban versus rural), individual preferences, available resources, and patient needs for tailoring of exercises, more supervision, and/or socialization. While the costs for exercise instruction per participant are generally lower for class-based exercise, both class-based and home-based programs have other costs to consider. Class-based programs require space, and patients will bear cost of travel and time for transportation to the class, while home-based programs will bear the travel and time costs for transportation to patients’ homes. Both programs may require equipment.

Implementers should also consider the trade-offs between patient adherence and program intensity. More frequent and longer duration exercise interventions are important for reducing fall rates, but may also decrease adherence and willingness to participate (Weerdesteyn 2006). Programs will waste resources if participants do not exercise or drop out. Sherrington’s meta-analysis suggested that the greatest effects on fall rates were from programs that included a minimum dose of 50 h of exercise (Sherrington et al. 2011; Sherrington et al. 2008). Achieving 50 h of exercise is possible through both a class such as a Tai Chi program, or a home-based option such as the Otago Exercise Program. Strategies for promoting adherence include regular phone calls, refresher sessions or home visits. Even with good adherence rates, programs will need to replenish their sample of participants on a regular basis due to attrition for health reasons.

Although existing studies elucidate the role that the health care setting may play in connecting older adults with exercise programs, several gaps remain. First, how the PCP should be involved in determining an older person’s appropriateness for exercise, both initially and over time, has not been specified. Evidence shows that PCPs are important for identifying and referring patients to exercise programs and the Stopping Elderly Accidents, Deaths & Injuries toolkit, developed by the Centers for Disease Control and Prevention, provides tools to help PCPs assess fall risk, educate patients, and select interventions (Centers for Disease Control and Prevention, 2015). Second, how exercise programs should be monitored to ensure that they meet minimum quality standards remains unknown (Tinetti et al. 2012). Third, it is unclear how exercise should be integrated with other fall prevention interventions. The Patient-Centered Outcomes Research Institute has funded a pragmatic multicenter trial of a fall prevention program (Trial Identifier: NCT02475850; www.stride-study.org) in which patients are being recruited from healthcare delivery systems; completion of this trial may address some of these knowledge gaps.

Our review has several limitations. First, we included only English-language studies. Second, we identified our source articles through existing systematic reviews of fall prevention and therefore may not have captured the most recent studies. Third, although we identified additional related papers to collect information on implementation, we are limited to what is reported in the studies. Therefore, we erred on the side of inclusion and provided findings even when they were based on a few studies. These limitations are mitigated by our detailed collection and synthesis of data from research that showed extensive variation in approach, thus increasing the likelihood that relevant themes were captured across a spectrum of programs germane to policymakers and implementers.

## Conclusions

In conclusion, building on strong evidence that fall prevention programs focused on exercise can help prevent falls, we have provided more detailed strategies to help clinical and health system leaders integrate fall prevention programs with routine care. Such strategies, however, require that PCPs receive infrastructure support to identify patients who may benefit from an exercise program, and to refer patients to programs that meet clinical needs and patients’ preferences.

## Abbreviations

PCP, primary care provider; USPSTF, U.S. Preventive Services Task Force.
